# Monolayer whole adherent islets: A novel tool for studying drug-induced diabetic phenotypes *in vitro*

**DOI:** 10.1371/journal.pone.0325421

**Published:** 2025-06-03

**Authors:** Makiko Kumagai-Braesch, Ming Yao, Nils Ågren, Ahmad Karadagi, Bo-Göran Ericzon, Anna Domogatskaya

**Affiliations:** 1 Division of Transplantation Surgery, Department of Clinical Science, Intervention and Technology, Karolinska Institutet, Stockholm, Sweden; 2 Department of Transplantation Surgery, Karolinska University Hospital, Huddinge, Sweden; NYU Grossman Long Island School of Medicine, UNITED STATES OF AMERICA

## Abstract

To understand the mechanisms of diabetes and develop novel drugs, various animal diabetes models as well as *in vitro* experimental systems have been established. Using isolated islets *in vitro* is associated with several problems: (1) discontinued blood microcirculation causes ischemia and central necrosis in isolated islets, (2) dissociation of islets into single cells reduces the total number of cells and impairs endocrine function. Here, we aim to establish a novel experimental method using isolated islets to characterize cell physiology and drug effects. We have developed a method to flatten islets into monolayers by culturing small, whole, non-disrupted mouse islets on laminin-521. In this culture, islet cells remain intact, and cell-cell junctions are not broken mechanically. Small mouse islets were cultured in a 96-well plate coated with laminin-521 for six days and then exposed to various concentrations of streptozotocin (STZ) for 24 hours. Monolayer islets exposed to 0.6 mM STZ-induced a metabolic diabetes-like phenotype, i.e., islet cell death, predominantly of beta cells, causing a shift in alpha-to-beta cell ratio. The stimulation index in a glucose-stimulated insulin secretion assay was reduced in STZ-treated islets. However, insulin production per beta cell did not change significantly. The monolayer whole islet assay format allows the extraction of quantitative data regarding endocrine cell populations, “endocrine sensitivity” (stimulation index), and “endocrine power” (amount of hormone secreted per cell). It is a novel, versatile, user-friendly, semi-automated, and financially attractive experimental platform.

## Introduction

Diabetes is a large and growing medical problem worldwide. In 2021, there were 537 million adult diabetes patients, and by 2045, this number is expected to exceed 783 million [1]. The majority of diabetic patients suffer from type II diabetes (T2D). These patients still have insulin-producing β-cells in their pancreatic islets. However, either insulin production in response to glucose is compromised, or the sensitivity of insulin in peripheral tissues becomes low (insulin resistance). The incidence of T2D is increasing due to aging and is closely related to increasing rates of obesity [2]. About 5–10% of diabetes patients suffer from type I diabetes (T1D), which is mostly diagnosed in children and young people. T1D is associated with the loss of β-cells in pancreatic islets. Diabetes complications over time can be severe, resulting in renal disease, cardiac disease, limb amputation, blindness, and chronic ulcers [3]. Furthermore, various drugs, such as steroids and calcineurin inhibitors, are known to be diabetogenic [4].

Research aimed to identify factors that cause diabetes, and new approaches to diabetes prevention and better strategies to cure diabetes are expanding [5–8]. Several animal models of diabetes have been established for use in diabetes research [9]. These animal models have been important in discovering anti-diabetic drugs, and their usage will continue and grow along with the expansion of diabetes research unless there are alternative non-animal research models with comparable biological relevance. Diabetic experimental animals frequently and inevitably endure stress, suffering, and poor life quality. To reduce animal suffering and obtain reliable data, it is important to establish *in vitro* research models that can replace some animal experiments with *in vitro* experiments.

Pancreatic islets are complex microanatomical structures, and this complexity is essential for their function [10]. Islets, comprising α-, β-, δ-, PP-, and ε-endocrine cells, are compact, self-regulated endocrine hubs that govern metabolism. Islet endocrine cells are not only neighbors; they are also principal coworkers that balance and fine-tune metabolism. Glucagon-secreting α- and insulin-secreting β-cells act as antagonists in controlling blood glucose levels, with δ-cells helping to regulate their activity. Shifts in proportion between α- and β-cells can cause abnormal changes in metabolic activity, while contact between endocrine cells is important for cross-regulation [11]. To understand and intervene in maintaining endocrine cell network function, it is important to establish a reliable *in vitro* culture system.

There are several *in vitro* research alternatives to animal models in islet/diabetes research. Most utilized methods fall into three major groups: (i) whole intact isolated islets, (ii) islet cell cultures derived from dissociated isolated islets and structures formed from such dissociated cells (islet sheets, 3D-organoids) and (iii) islet cell cultures of altered nature, such as immortalized islet cell cultures or embryonic stem cell (ESC)/induced pluripotent stem cell (iPSC)-derived islet-like cells and three-dimensional cell arrangements. However, each of these methods has considerable disadvantages. Whole isolated islets in group (i) vary in size and shape, and once they are disconnected from their microcirculatory environment, ischemia may cause central necrosis, causing issues for them as a consistent and relevant research model [12,13] (S1A Fig). In group (ii), the procedure of cell dissociation using a proteolytic enzyme causes massive cell loss, functional loss, and architectural disruption [14]. In group (iii), these cells are artificially engineered objects and may not be suitable for examining the natural character of pancreatic islets, though they are immortalized cell lines, for instance, mouse insulinoma MIN6 immortalized β-cell line [15], and stem cell-derived pancreatic islet cells successfully imitate some properties [16,17].

Extracellular matrix molecules, especially islet-specific laminin isoforms, play an essential role in pancreatic islet structure and function. Laminins are large extracellular matrix molecules that communicate with cells, convey specific signals, and thus actively modulate their behavior. [18]. Although the known 16 laminin isoforms appear relatively similar, they may influence associated cells in diverse, sometimes opposite, ways. [18,19]. Laminins are trimeric molecules; every isoform is attributed by three numbers, starting with the number of an α-chain (five α-chains existing, α_1–5_), a β-chain (β_1–4_), and a γ-chain (γ_1–3_); for example, laminin-521 used in our work is composed of α_5_-, β_1_- and γ_1_-chains. As shown in [15], laminins with α_5_- and α_4_-chains are essential to natural islet β-cell niches and support β-cells functionally.

Shimizu et al.[20] have shown that laminin-332 could support the β-cell sheet and maintain insulin release after the dissociation of cells from islets; this fabricated cell sheet model composed of dispersed islet cells has been further evaluated by Saito et al. [21] and Nagaya et al. [22]. It has been shown that pancreatic islet cells within islets readily adhere, flatten, and spread out on laminin-511 or laminin-521-coated surfaces [23], resulting in issued patents, US Patent 9499794 [24] and US Patent 11155781 [25]. This model has proven to be physiologically relevant since it successfully cures diabetes in streptozotocin (STZ)-treated mice, with a stable effect lasting for 2 months [26]. The effect of islet flattening on laminin-521-coated surfaces, well-validated by Sigmundsson [26], partially alleviates the effect of central ischemia and necrosis. It is achieved by non-compulsory reformation of whole islets based on their natural high affinity to laminin-521, wherein functional islet cell-cell junctions are not broken through force.

The adherent, flattened islet approach appears to be an attractive platform for designing a 3R alternative. However, there are challenges. When large and medium-sized islets are flattened on laminin-521, the spreading occurs only at the periphery of the islets. At the same time, the middle part remains bulged for several days and is prone to central necrosis (S1B Fig). Necrotization within isolated islets significantly reduces their relevance as research models. As whole pancreatic islets are isolated from mouse pancreases by enzymatic digestion, in case of incomplete digestion, some isolated islets may have non-islet cells attached to their surface [13], which may spread in a fibroblast-like manner and vigorously proliferate, causing contamination (S1C Fig). Though islets spreading on laminin-521 become flat, they still consist of multiple cell layers (S1D Fig). Such an arrangement may cause uneven distribution of drugs, which may compromise experimental data.

In order to overcome the limitations of the flat islets, as mentioned above, we established a new method of monolayer whole adherent islet culture that allowed for the development of a reliable and user-friendly flat whole islet model for *in vitro* research to quantitate and characterize endocrine cells. Using this model, we analyzed the quantitative dose-dependent effect of STZ on *in vitro* islet culture endocrine cell numbers, glucose sensitivity, and “endocrine power.” STZ was first described as an antibiotic in 1959 [27], and had been well characterized as a substance that causes diabetes by damaging β-cells in pancreatic islets *in vivo* [28–31].

The individual aims of our study were the following:

1. Develop a model of flat adherent islets cultured on laminin-521, suitable for *in vitro* semiautomated assays, providing reliable quantitative data regarding cell populations and endocrine function.
2. Explore the possibility of direct and unimpeded delivery of experimental substances dissolved in a culture medium uniformly to all the islet cells.
3. Explore the possibility of inducing diabetes in flat islets *in vitro* by treating them with STZ and estimate the effective range of STZ dose, effects of STZ on endocrine islet cell populations, the ability of β-cells to release insulin in a glucose-dependent manner, and the “endocrine power” of β-cells (amount of insulin released per β-cell per hour when stimulated by high glucose concentration).

## Materials and methods

### Animals

#### Mouse facility.

Male Balb/c (H-2^d^) mice were purchased from Charles River Inc. (Sulzfeld, Germany) and used as donors of mouse pancreatic islets. The mice were in a pathogen-free facility at Karolinska Institutet, Huddinge, Sweden. The experiment was approved by the Swedish Board of Agriculture (Dnr. 78-15 and 5.2.18-11712/14). The study was conducted according to the guidelines for using Laboratory Animals at Karolinska Institutet, Sweden.

#### Humane endpoints.

The mice had free access to food and water. The animal’s condition was observed daily by trained personnel in the animal facility at Karolinska University Hospital Huddinge, Sweden. If an animal shows signs of bad general condition, it will be subjected to euthanasia according to ethical permission (Dnr. 78-15 and 5.2.18-11712/14). After one to four weeks of observation at the animal facility, the mice were euthanized by neck dislocation, and the tissues were procured for this study according to the method below.

### Islet culture, STZ treatment, and glucose-stimulated insulin secretion assays (GSIS)

#### Mouse pancreatic islet isolation.

The pancreatic islets from 20 Balb/c mice (males 12–15 weeks) were isolated as previously described [32,33]. After gradient purification, islets were handpicked and sorted by size. Larger islets (diameter >50 µm) were used in pancreatic islet transplantation in a different study [34], while islets that were too small for transplantation were further purified and used in this study.

#### Islet handpicking and selection.

The islets, isolated according to the above procedure, were handpicked 4–5 times. The islets were kept in the cell culture incubator at 37°C and 5% CO_2_ in excessive volumes of islet culture medium, comprising 44% RPMI-1640 medium (Invitrogen), 43% CMRL-1066 medium (Invitrogen), 10% fetal bovine serum (Fisher Scientific), 10 mM HEPES (Invitrogen), 1% GlutaMax (Invitrogen), 1% Penicillin-Streptomycin (Fisher Scientific). The procedure was performed under an inverted microscope (Olympus CKX41 Microscope) at 4x, 10x, and 20x magnifications to evaluate the quality and purity of selected islets. The islets were cleaned from non-islet cells floating in suspension and sorted by size. A group of islets of small size, ranging from 50 to 130 μm in diameter (mean of 90 μm), were used in this study. Islets with signs of central necrosis, with non-islet structure attached, non-round shape, and sizes below 50 μm or over 130 μm in diameter were discarded from the study. The diameter of the islets was measured by the Olympus Ix70 inverted fluorescence and phase contrast tissue culture microscope using CellSens software (Olympus).

#### Islet source: leftover material from transplantation studies.

In most experiments, we used islets that were preclinical-oriented leftovers from islet transplantation experiments. For our research, islets 90 μm in diameter were most desirable; they were often left unused from the transplantation experiments since they contained too little cell material. This consideration is beneficial for the 3R purposes since no or only a few extra mice are euthanized to produce the islets for *in vitro* studies.

#### Plating islets on laminin-521 coated plastic plates.

For high-quality 3D spinning disc microscopy, immunofluorescent islet cell imaging, 96-well culture plates with ultra-thin, flat, transparent ultra-low plastic bottoms were used (Perkin-Elmer, CellCarrier™-96 Ultra, # 6055300). Laminin-521 (BioLamina, Sweden) frozen solution was thawed at +4°C and diluted in +4°C cold PBS buffer to 14 μg/ml. The plates were coated with laminin-521 solution in PBS (80 μL/well) for at least 16 hours at +4°C (alternative method: 2 hours at +37°C) and washed with PBS buffer 3 times before use to remove the unbound laminin and sodium azide, a toxic preservative in the laminin commercial solution.

#### Islet adherence to laminin-521 coated plates.

Eight hand-picked islets were plated onto laminin-521-coated plates per well. The islets were cultured at 37°C, 5% CO_2_ in a humidified atmosphere, in the islet culture medium (described above), with a volume of 200 μL/well. The culture medium was filtered prior to use to remove serum precipitates that may complicate the analysis of the immunofluorescent imaging data later. Empty plate wells neighboring the islet culture were filled with 300 μL of PBS buffer to prevent medium evaporation. The islet culture was kept intact for 3 days to allow the islets to attach and spread on the laminin-coated plastic surface. After that, the medium was changed 3 times a week, every 2–3 days.

#### STZ preparation.

Dry STZ (Sigma-Aldrich) was diluted in water to make a concentrated stock solution with a concentration of 50 mM (13,2 mg per 1 ml of cold water). The stock solution was kept on ice to prevent loss of STZ activity. It was diluted in a culture medium to the final concentrations of 0.6, 0.2, 0.06, and 0.02 mM. The STZ-containing medium should be added to the islet culture immediately after preparation to maintain the activity of STZ.

In our assay, incubation with 1 mM STZ solution for 24 hours caused almost complete loss of cells; therefore, we investigated the effect of lower dosages of STZ, as shown above. Islets, untreated with STZ, served as a control.

#### STZ treatment.

STZ was applied to the flat adhered islets 7 days after the islet plating, which is sufficient for the islets to acquire a monolayer shape and thus allow direct instant access of STZ from the culture medium to all the islet cells. The islets were cultured in the presence of STZ-containing medium for 24 hours, then washed 3 times with culture medium and cultured as previously before the treatment.

#### GSIS assays.

Two GSIS assays were performed, one day before and six days after the STZ treatment, to compare the data and assess the effect of STZ on the islets’ ability to produce insulin in a glucose-sensitive way. We used a culture medium (“GSIS-medium”) comprising 96% RPMI-1640 without glucose (Invitrogen), 1% FBS, 10 mM HEPES, 1% GlutaMax, 1% Penicillin-Streptomycin with the addition of D-glucose (Invitrogen) to concentrations of 2 mM (low glucose level) or 25 mM (high glucose level).

The islets were first incubated with a fresh culture medium for 1 hour, then replaced with 100 μl of GSIS-medium containing 2 mM glucose (low level). After 1 hour of incubation, the “low glucose” medium was collected into tubes, and the wells were incubated with 100 μl of GSIS medium containing 25 mM glucose (high level), which was also collected after 1 hour. The collected medium was centrifuged in a microcentrifuge (Himac CT15RE, Hitachi Centrifuges) for 4 minutes at 4000 rpm at 4°C, and the supernatant was frozen and stored at −20°C for subsequent ELISA analysis.

#### ELISA.

The mouse insulin levels in the culture medium collected from the GSIS assay were measured by insulin ELISA kits (Mercordia, Uppsala, Sweden). The procedure has followed the manufacturer’s recommendations. In brief, the samples were kept frozen at −20°C until use. 10 µl of each sample was plated (it was diluted 10 times with dilution buffer if necessary) on the anti-insulin antibody, pre-coated plate, and the enzyme-conjugated secondary antibody, and assay buffer were mixed according to the protocol. The plates were incubated at room temperature on a plate shaker for two hours. After washing the solution, TMB substrate was added. The reaction was stopped 10–15 minutes after incubation by adding a stop solution. Absorbance was measured at 450 nm by spectrophotometer.

Two output parameters were used to characterize islet insulin-producing function: (i) Stimulation Index, SI (“endocrine sensitivity”), and (ii) average β-cell efficacy (“endocrine power”): the amount of insulin, secreted in response to high glucose concentration stimulation, per β-cell per hour.(See below Calculations and formulas)

The next day after the GSIS test, the islets were fixed, and immunocytochemistry staining was performed. α-cells, and β-cells were quantitated as described below.

### Immunocytochemistry, 3D microscopy, and cell quantification

#### Cell fixation.

The islets were washed twice and fixed by adding a 4% paraformaldehyde (PFA) solution in PBS buffer, pH 7.4, at room temperature for 20 minutes. Subsequently, the wells were washed with PBS buffer 3 times and stored in PBS at +4°C, sealed with parafilm to prevent evaporation.

#### Immunostaining.

For fixed islet cell permeation, the samples were treated with 0.1% Triton-X in PBS buffer for 15 minutes at room temperature and then washed twice with PBS. The samples were incubated with the blocking buffer (10% fetal bovine serum, 0.5% Tween-20 in PBS buffer) for 30 minutes at room temperature (alternative: overnight at +4°C) to block nonspecific bindings of the antibodies. The samples were stained, subsequently, by primary mouse-anti-mouse Glucagon antibody (K79bB10, mouse IgG1, Abcam, 1:500 in the blocking buffer), washed 3 times by the washing buffer (1% bovine fetal serum, 0.5% Tween-20 in PBS, pH 7.4), then stained by secondary donkey-anti-mouse antibody Alexa-647 coupled (polyclonal antibody, Invitrogen), diluted 1:200 in the blocking buffer, washed 5 times with the washing buffer. Then the samples were stained by FITC-coupled mouse-anti-mouse/human insulin antibody (2D11-H5, mouse IgG1, MabtechAB, Nacka, Sweden), diluted 1:50 in the blocking buffer, incubated 2 hours at room temperature (alternative: overnight at +4°C), washed twice, stained with DAPI (Invitrogen) 1:1000 in washing buffer for 30 minutes, washed 3 times with the washing buffer and 3 times with PBS. The samples were stored in PBS at +4°C and sealed with parafilm.

#### Positive and negative control.

For the positive staining control, we use the antibody-stained flat adherent healthy islets that were not treated with STZ. For the negative staining imaging control, we used flat adherent healthy untreated islets stained by the same scheme, whereas antibodies were replaced by immunoglobulin from the same species.

#### Storage.

The stained plates were stored in PBS at +4°C and sealed with parafilm to prevent evaporation. They were pre-warmed to room temperature 30 minutes prior to imaging.

#### Spinning disk 3D microscopy.

The 3D imaging of the immunostained adherent islet culture was performed in the Live Cell Imaging Facility, Karolinska Institutet, Sweden, using the spinning disc mode on the Nikon Eclipse Ti confocal microscope. The ultra-thin, ultra-low bottom plates specifically designed for fine microscopy (#6055300, PerkinElmer, US) were necessary to get quality images. PFS (Perfect Focus System by Nikon, Japan), a system that automatically adjusts confocal focus to the local Z-level to the plate surface, was used to keep the islets in focus. Confocal image acquisition was performed at 20x magnification since 10x allows lower quality and 40x results in unacceptably long timing and enormous files (since we aim to capture not a few but 200 islets in the 3D mode in one session). The software for capturing images was NIS-Elements (Nikon, Japan).

**Fig 1:**
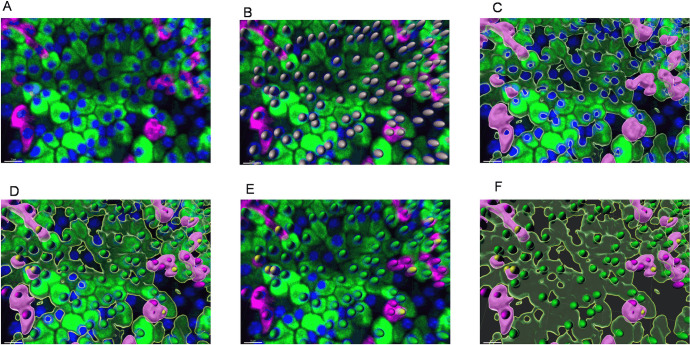
The procedure of quantification of α-, β- and total islet cells from 3D imaging of immunostained monolayer whole islets. (A) The original 3D image of a local segment of an immunostained islet. The image was captured by the 3D spinning disc technique on a Nikon Eclipse Ti confocal microscope, at 20x magnification. The Z-stacks were captured with a step of 2 μm. Each islet was captured individually. The 3D images of the islets were analyzed by Imaris software. Blue: DAPI staining, identifies all the cell nuclei; Green: insulin immunostaining (cytoplasm); Pink: glucagon immunostaining (cytoplasm). Bar size = 15 μm. (B) Mathematically identified cell nuclei overlayed upon the original 3D image of the immunostained islet. The white ellipsoids are mathematical objects that represent DAPI-positive nuclei of all cells. Since the islet culture is not contaminated by non-islet cells, all the identified cells are considered to be islet cells. Bar size = 15 μm. (C) Mathematically identified α- and β-cell cytoplasm objects overlayed upon the original 3D image of immunostained islet. The green semitransparent surface object represents insulin-positive cytoplasm, and the pink opaque surface object represents glucagon-positive cytoplasm. Bar size = 15 μm. (D) Mathematically identified α- and β-cell nuclei objects overlayed upon mathematically identified α- and β-cell cytoplasm objects and the original 3D image of immunostained islet. Green ellipsoids represent β-cell nuclei objects adjacent to the green insulin-positive cytoplasm object. Pink ellipsoids represent α-cell nuclei objects adjacent to the pink glucagon-positive cytoplasm object. Yellow ellipsoids represent the nuclei that were at the first attributed to insulin-positive by the automatic program, and then the attribution was corrected from β-cell to α-cell by an extra step (automated). Bar size = 15 μm. (E) Mathematically identified α- and β-cell nuclei objects overlayed upon the original 3D image of immunostained islet. This optional step allows manual inspection of the automated attribution of the nuclei. Bar size = 15 μm. (F) Mathematically identified α- and β-cell nuclei objects overlayed upon mathematically identified α- and β-cell cytoplasm objects. The original 3D image is removed from the view. Those objects are used by Imaris software to generate quantitative data regarding cell and cell populations from 3D images of adherent islets. Bar size = 15 μm.

The confocal microscopy imaging was performed in three steps. Step 1, the complete screening of all the stained wells on the whole plate was performed in automatic 2D imaging mode using one channel (DAPI) only to identify the location of all the islets. The PFS mode kept the islets always in focus by automatically correcting for the culture plate bottom Z-position deviations. In step 2, the precise X, Y, and Z locations of the adherent islets were identified and marked for the subsequent automatic imaging (Step 3). It was the only part of the process that required manual management; however, it could be performed in 30 minutes for 200 islets using NIS-Elements software. Step 3, the 3D images of the individual islets were acquired in all the color channels: Alexa-647 (deep red), FITC (green), and DAPI (blue) (example: see Fig 1A). We have previously successfully implemented and described a similar approach with the same equipment and software for imaging and analyzing immunostained tissue slices with transplanted islets [35], i.e., first, large surface screening in 2D mode for one color channel to localize objects of interest; second, marking locations of those objects for the 3D image capturing; third, capturing 3D images with multiple color channels.

### 3D Image analysis and cell quantification

#### Imaris: software for the identification and quantification of 3D objects of complex shape.

The analysis of the 3D islet images (Fig 1A) was performed by Imaris v.8 software (Bitplane, Oxford Instruments), an approach similar to that described previously [35]. The software is well-verified as a cell quantification program [36]. We used it to (i) identify all the cells by DAPI-positive nuclei (the software marks the identified nuclei as “spheres” object Fig 1B), (ii) identify the insulin-positive (FITC, green) and glucagon-positive (Alexa-647, pink) cytoplasm that due to complex geometrical shapes are identified as “surfaces” objects, Fig 1C), (iii) identify the nuclei (“spheres” objects) within user-defined proximity (4 μm/ 2 μm, as will be described later) to either glucagon-positive (pink) or insulin-positive (green) cytoplasm (“surface” objects); thus attributing them as α- or β-cells (Fig 1D). The algorithms of nuclei and cytoplasm identification rely on fluorescence intensity, geometrical proportions of the objects and principle of cohesiveness of the objects.

#### Process of automated batch analysis.

Since there is always a variation in positive vs. background immunostaining intensity levels between different plates, even if they were stained according to the same protocol, the intensity threshold parameter and, possibly, other parameters must be adjusted manually for each particular experimental plate. There are five pipelines to be followed: (I) adjusting intensity parameters using several positive (stained with specific antibodies) and several negative controls (stained with relevant immunoglobulins instead of antibodies), (II) running a small-size batch automated analysis for the selected controls; checking that α- and β-cells are detected correctly in all the positive controls, and no α- or β-cells are detected within the negative controls, (III) running full-size batch automated analysis for all the samples, including positive and negative controls, (IV) manually checking that identification of α- or β-cells appears to be correct, (V) extracting the data for statistical analysis.

#### Process of cell identification: the total amount of islet cells ($N_{total}\;$), β-cells ($N_{\beta }\;$), α-cells ($N_{\alpha }$).

Islet cells are identified by Imaris software according to the following protocol:

(1) all the islet cell nuclei ($N_{total}\;$) by the “spots” (spheres) protocol, using DAPI (blue) channel (intensity threshold defined manually), see Fig 1B.
(2) β-cell cytoplasm outer surface, based on insulin staining intensity (threshold defined manually), is identified as “β-surface”, (Fig 1C green area)
(3) α-cell cytoplasm outer surface, based on glucagon staining intensity (threshold defined manually), is identified as “α-surface”, (Fig 1C pink area).
(4) β-proximal-4 μm spheres are identified as spheres that are 4 μm or closer to the “β-surface”, (Fig 1D green nuclei).
(5) nonβ-α-proximal-4 μm spheres are identified as non-β spheres that are 4 μm or closer to the “α-surface”, (Fig 1D pink nuclei).
(6) extra correction step: β-to-α correction-2 μm spheres are identified as β-proximal-4 μm spheres that are 2 μm or closer to the “α-surface”. β-cells ($N_{\beta }\;$) are identified as β-proximal-4 μm spheres minus β-to-α correction-2 μm spheres. α-cells ($N_{\alpha }$) are identified as α-proximal-4 μm spheres plus β-to-α correction-2 μm spheres (Fig 1D yellow nuclei).

Spheres and surfaces are identified in automatic batch analysis mode enabled by the Imaris software. After that, the results can be manually examined to see if automatically identified “spheres” and “surfaces” properly relate to the actual 3D image. An extra correction step may be added manually to correct the automated identification of β- and α-cells (including the automated β-to-α correction step) (Fig 1E). After the manual correction, the number and area of each type of cell are calculated (Fig 1F).

#### Calculations and formulas.

The raw data is retrieved from the two GSIS assays (before and after STZ treatment) and islet cell population analysis from the 3D images analyzed by Imaris software (total islet cell population ($N_{total}$), β-cells ($N_{\beta }\;$) and α-cell number ($N_{\alpha }$)).

The α-to-β cell ratio is defined as: ($\frac{N_{\alpha }}{N_{\beta }}\;$).


$$\;SI=\frac{Insulin\;(high\;glucose=25\;mM)}{Insulin\;(low\;glucose=2\;mM)}$$

Change in SI due to STZ treatment is defined as:


$$Change=\frac{{SI}_{after\;STZ\;treatment}}{{SI}_{before\;STZ\;treatment}}$$

The ability of β cells, on average, to secrete insulin when stimulated with high glucose levels (“endocrine power”) is defined as: $Efficacy=\;\frac{Insulin\;secreted(glucose=25\;mM\;pg}{N_{\beta }\;\cdot \;incubation\;assay\;duration(=1\;hour)}$, where Insulin secreted (pg)= Insulin concentration pg/mL(by ELISA)· assay volume .

### Statistics

The values were shown as mean ± SD (n = 4). A Kruskal-Wallis test with Dunn’s multiple comparisons was used to compare values among different concentrations of STZ treatment groups. The Mann-Whitney test was used to compare the two groups. P < 0.05 is considered a significant difference.

## Results

### Monolayer whole adherent islets as a platform for a 96-well plate *in vitro* drug evaluation quantitative assay

**Fig 2:**
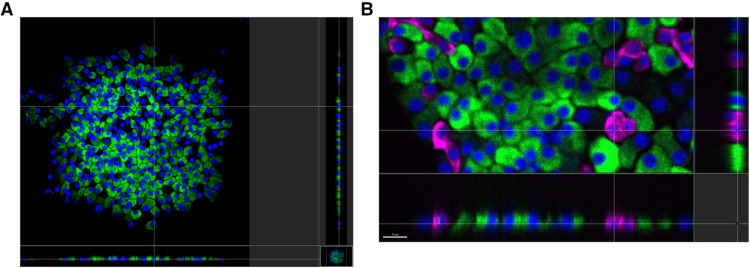
Monolayer whole adherent islets. 3D confocal images of immunostained islets are virtually dissected in three dimensions to confirm monolayer structure. (A) Adherent whole islets of small size (about 90 μm in diameter) were cultured for about two weeks according to the protocol described in Materials and Methods. The islets were immunostained (blue: DAPI, green: insulin antibodies), and 3D images were captured by spinning disc technique in a confocal microscope Nikon Eclipse Ti. Using Imaris software, the 3D images were virtually dissected in three planes. The XZ and YZ dissections (below and on the right of the XY dissection) allow us to confirm that the islet structure is truly monolayer. Bar size = 30 μm (B): A small fragment of an adherent monolayer whole islet 3D image (immunostaining shows blue: DAPI, green: anti-insulin antibodies, pink: anti-glucagon antibodies) is virtually dissected in three planes. The XZ and YZ dissections (below and on the right of the XY dissection) allow us to confirm that the islet structure is truly monolayer. Bar size = 15 μm.

We discovered that a population of small-size islets, on average 90 μm in diameter, spreads on laminin-521 coated plastic rapidly and evenly, forming monolayer-like structures within only a week (Fig 2A monolayer islets).

A 3D image of such a monolayer islet was taken using the spinning disc confocal microscopy technique. The software for analysis of 3D images (Imaris, Bitplane, Oxford Instruments) allows the virtual dissection of the islet in all three dimensions to be performed. As shown in Fig 2A and 2B, both vertical dissection images confirm that the islet is a monolayer and cohesive structure, making cell quantification more reliable.

Transformation of whole isolated islets into cohesive monolayer adhesive culture is achieved non-traumatically. The islets undergo slow and gradual deliberate reformation only due to the natural strong affiliation between the islet cells, and laminin-521 immobilized on the plastic surface. No trauma (mechanical or enzymatic) is inflicted on the islet cells or their cell-cell contacts.

The monolayer shape of the flat islets allows direct and instant access of the experimental molecules from the culture medium to all the islet cells at once. Thus, we could treat whole isolated islets *in vitro* with STZ, ensuring that all the islet cells receive the same dose, activity, and exposure time of the toxin.

Due to a careful islet selection protocol (see Materials and Methods), we achieved an experiment setup with 200 whole islets (25 plate wells, eight islets per well), which turned into a monolayer within a week and without contamination by non-islet cells fibroblast morphology. Each islet comprised a cohesive entity of compact cells with compact nuclei and cytoplasm stained positive for either insulin (β-cells) or glucagon (α-cells).

### Dose-dependent effect of STZ on endocrine cells by using a monolayer islet model: quantitative evaluation

**Fig 3:**
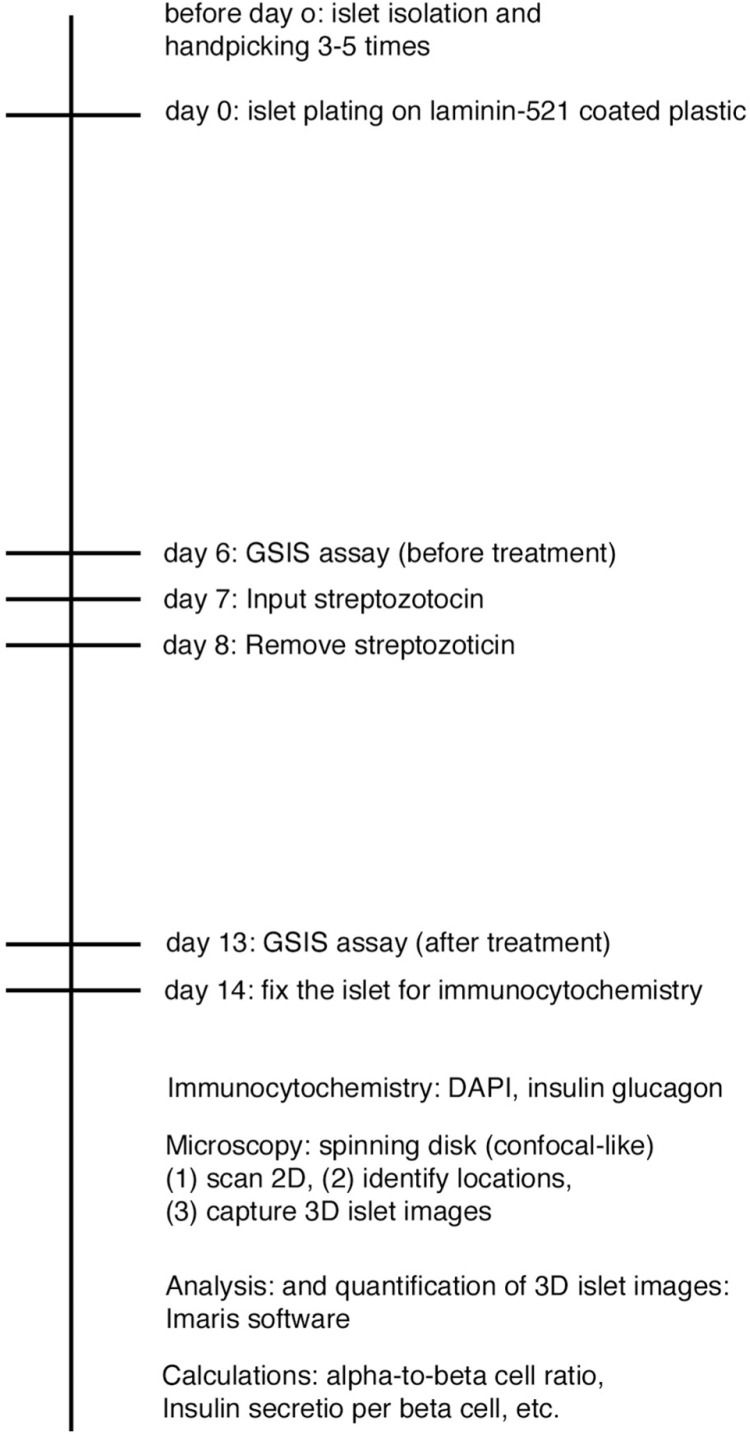
Experimental timeline for the streptozotocin-treatment experiment. Days 0-6: isolated islets of small size (about 90 μm in diameter) are cultured on laminin-521-coated plastic surface in 96-well culture plates aimed for fine microscopy. A week is sufficient to allow the islets to spread into a monolayer structure. Day 6: The first GSIS assay, prior to STZ treatment. Day 7: STZ treatment. STZ dosages: 0.6, 0.2, 0.06, 0.02 mM and untreated control. Day 8: STZ is removed. Day 8–13: Regeneration from STZ-induced damage. Day 13: The second GSIS assay, after the streptozotocin treatment and regeneration phase. Day 14: The monolayer islet culture is fixed.

Using the newly developed *in vitro* islet culture model, the dose dependence of STZ on endocrine islet cells was evaluated. Small-sized whole isolated islets were cultured on laminin-521 for one week to attain a monolayer form. GSIS assay, a glucose sensitivity and insulin productivity test, was performed 1 day before STZ treatment and repeated 6 days after STZ treatment. At the end of the experiments, the islet cells were fixed and immunostained for glucagon and insulin in the cytoplasm to identify α- and β- cells and also stained with DAPI to identify all the cell nuclei (Fig 3, islet culture experiment pipeline).

Within a month, the fixed islets are immunostained (for DAPI, insulin, and glucagon). 3D images of all islets are captured by the spinning disc method in 96-well plates with a confocal Nikon Eclipse Ti microscope. The 3D images are evaluated by Imaris software. The nuclei (of total cells, α-cell and β-cell) and the cytoplasm areas (of α-cell and β-cell) are identified using automated batch analysis mode, and the numbers of total cells, α-cell and β-cell are acquired based on this data.

Here, we demonstrated (1) a reduction in insulin secretion capacity and sensitivity, a toxic effect on endocrine cells, especially β-cells, and (2) endocrine cell population change, i.e., a shift in α-to-β cell ratio, and (3) the STZ treatment effect on the insulin secretion of a single β-cell level.

#### (1) β-cell ability to release insulin under glucose stimulation.

**Fig 4:**
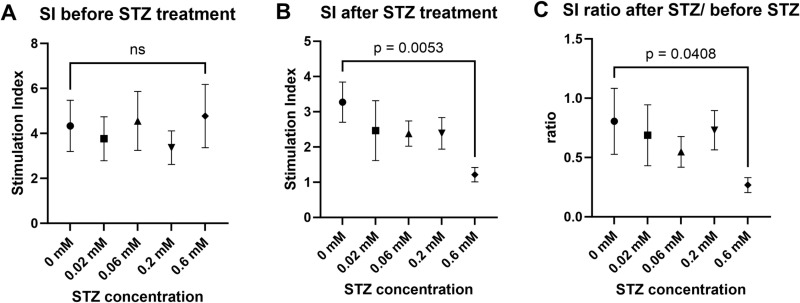
STZ treatment effect on SI in glucose-stimulated insulin secretion (GSIS) assay in monolayer adherent whole islets. (A) SI measured before dose-dependent STZ treatment (day 6 of the experiment) in the islet groups relevant to Fig 5B. Healthy monolayer flat islets demonstrated SI>>1 in all the experimental groups, and there was no statistically significant difference between the groups prior to STZ treatment. ns: not significant. (B) SI measured after 24 hours of dose-dependent STZ treatment (day 7–8) and STZ-free regeneration period (day 13). Though all the islet groups demonstrated SI > 1, islets treated with 0.6 mM STZ demonstrated a strong reduction in insulin reactivity (S) even after the recovery period. (C) Change of SI after STZ treatment: SI after STZ treatment and recovery (Fig 4B data), compared to SI just before the treatment (Fig 4A data). Change is calculated as the ratio of SI after the treatment to SI before the treatment. The islets treated with 0.6 mM STZ demonstrated a significantly stronger reduction in the ratio.

The GSIS assay performed 1 day before the STZ treatment has shown an SI average of 4.8 ± 2.9 (STD), > 2.4 in all the individual samples. This indicates that the flat islets in all the groups maintained a natural ability to release insulin in a glucose-dose-dependent manner (Fig 4A), and there was no statistically significant difference between the experimental groups before STZ treatment.

Six days after the STZ treatment, all the groups exhibited SI > 1, showing that the dose of STZ did not eliminate glucose sensitivity in the treated islets. However, the average SI in the group treated with 0.6 mM STZ was significantly lower than in the untreated group (Fig 4B). Change of SI, according to the formula: Change = SI_after STZ treatment_/ SI_before STZ treatment_, was compared between the groups of islets treated with 0.6, 0.2, 0.06, 0.02, and 0 mM (untreated) STZ. A clear decline in sensitivity was noted in the 0.6 mM STZ-treated group compared to the other groups (Fig 4C).

The effect of STZ in lower concentrations, such as 0.2, 0.06, and 0.02 mM, did not result in any statistically significant effects.

#### (2) Islet cell populations.

**Fig 5:**
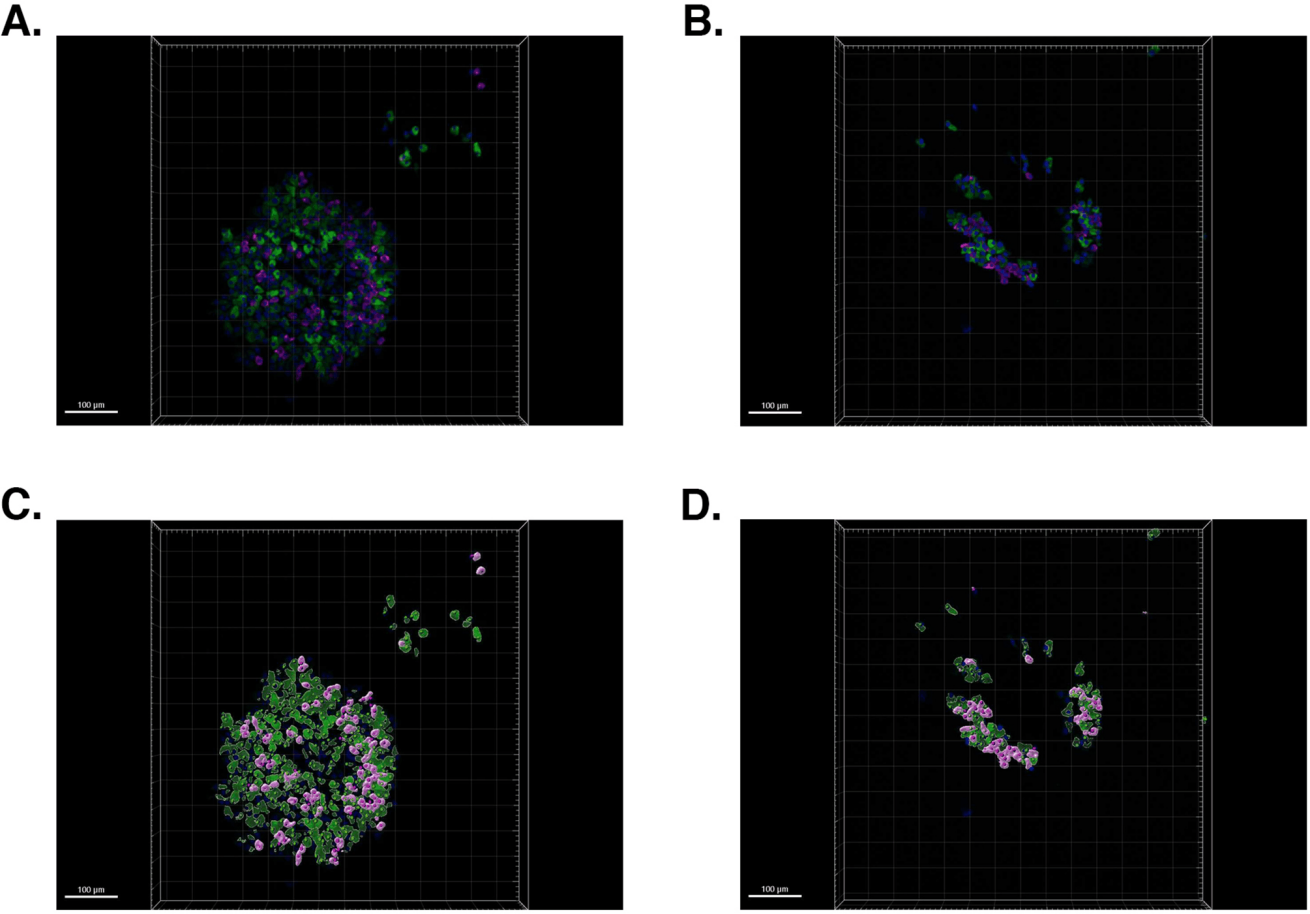
3D images of monolayer whole islets, untreated vs STZ-treated. The original images of immunostained islets captured by microscopy vs. calculated mathematical objects representing α- and β-cells. Monolayer whole adherent islets of small size, healthy untreated (left) vs. treated with 0.6 mM STZ (right) after a one-week recovery period were fixed and immunostained ((blue: DAPI, green: insulin antibodies, pink: glucagon antibodies). Top panel: 3D images (the top panel A and B) were captured using the spinning disc technique by a confocal microscope Nikon Eclipse Ti. Bottom panel (C and D): Using Imaris software, 3D mathematically modeled objects were built, representing: α- cell glucagon-positive cytoplasm area (pink opaque surface of complex shape), β-cell insulin-positive cytoplasm area (green semitransparent surface of complex shape), α-cell nuclei (pink and yellow ellipsoids), β-cell nuclei (green ellipsoids) Bar size = 100 μm. (A) Untreated healthy monolayer whole islet, the original 3D image; (B) Monolayer whole islet treated by 0.6 mM STZ after recovery, the original 3D image; (C) Untreated healthy monolayer whole islet, mathematically calculated 3D objects representing: α-cell cytoplasm (pink), β- cell cytoplasm (green), α-cell nuclei (pink dots), β-cell nuclei (green dots). (D) Monolayer whole islet treated with 0.6 mM STZ after recovery, mathematically calculated 3D objects representing: α-cell cytoplasm (pink), β-cell cytoplasm (green), α-cell nuclei (pink dots), β-cell nuclei (green dots).

**Fig 6:**
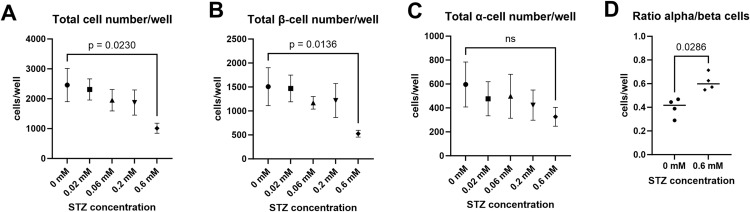
STZ dose-dependent effect on endocrine islet cell populations. (A) Total cell population (***N***) per experimental well was measured based on 3D identification of DAPI-positive (blue) cell nuclei, (B) β-cell population ($N_{\beta }\;$) per experimental well, was measured based on 3D identification of the cell nuclei proximal to insulin-positive (FITC, green fluorophore) cytoplasm, (C) α-cell population ($N_{\alpha }$) per experimental well, was measured based on 3D identification of the cell nuclei proximal to glucagon-positive (Alexa-647, pink fluorophore) cytoplasm, ns: not significant. (D) α-to-β cell ratio was measured as a ratio between α-cell population ($N_{\alpha }$) and β-cell population ($N_{\beta }\;$). The values between the groups of STZ 0mM and STZ 0,6 mM were compared.

At the end of the assay, the cultured islets were fixed, and cell numbers (total islet cells, α- and β- endocrine cells) were acquired, as described in Materials and Methods. Typical examples of stained cells are shown in Fig 5. Adherent flat islet cell number per well was counted as DAPI-positive cells. Distinct, statistically significant reduction of the total islet cell population (N_total_) and β-cells (N_β_, insulin-positive cells) (Fig 6A, 6B) was noted in the group treated with 0.6 mM STZ for 24 hours compared to the control group (untreated islets). The total cell number $N_{total}\;$reduced several times (Fig 6A). The β-cell number $N_{\beta }$ also reduced several times (Fig 6B). There was no significant reduction in α-cell number $N_{\alpha }$ (Fig 6C). The α-to-β cell ratio ($\frac{N_{\alpha }}{N_{\beta }}\;$) has increased significantly (Fig 6D). The effect of STZ in lower concentrations, such as 0.2, 0.06, and 0.02 mM, did not result in any statistically significant effects.

#### (3) Amount of insulin secreted per β-cell under stimulation with high glucose.

**Fig 7:**
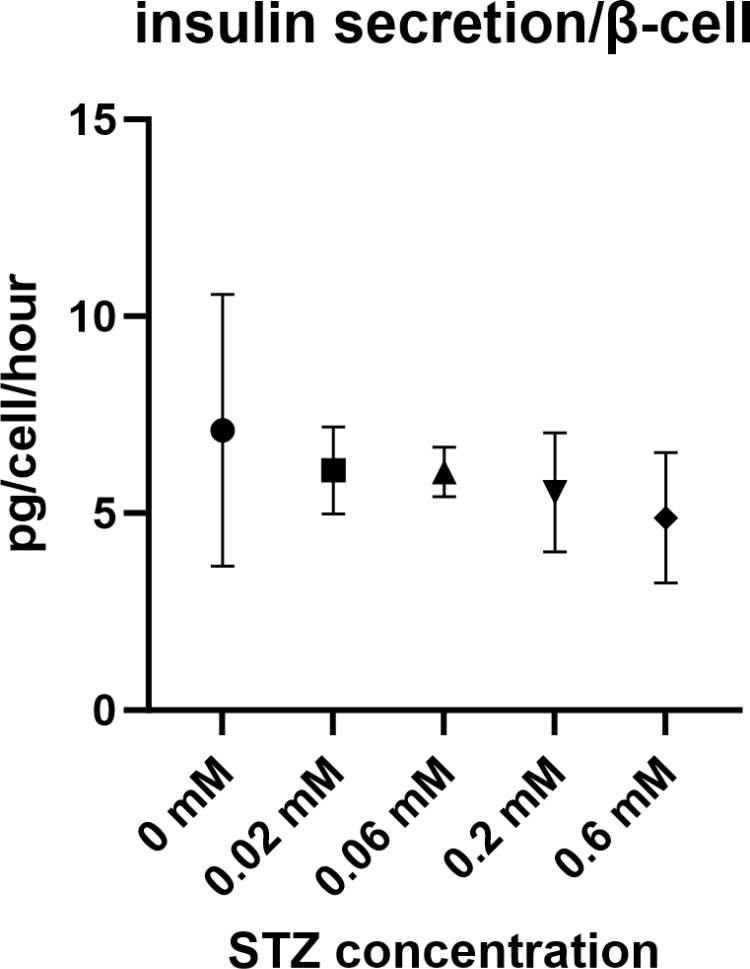
STZ dose-dependent effect on insulin function in adhered monolayer islets. The efficacy of average β-cell (so-called “endocrine power”) was evaluated as the amount of insulin produced per average β-cell per hour when stimulated with a high 25 mM glucose concentration. It was measured as the amount of insulin produced by all islets (stimulated) within a well of the culture plate (concentration multiplied by volume), divided by the number of β-cells in the well (Fig 6B), and incubation time (one hour). No significant differences among groups were observed.

To evaluate the amount of insulin that can be secreted by an average β-cell responding to high glucose stimulation (a parameter that can be called “endocrine power”), we have divided the amount of insulin produced per well at high glucose levels per β-cell number per well ($N_{\beta }$), per exposure time. In untreated islets, β-cells secreted about 6–7 pg/β-cell/hour. The insulin secretion by an average β-cell when stimulated by high glucose concentration did not change significantly by STZ treatment (Fig 7).

## Discussion

### Main findings

In this study, we have established a novel method of creating a monolayer whole adherent pancreatic islet organoid culture system *in vitro*, achieved by non-traumatic reformation of whole islets through adherence to the natural ligand laminin-521. We demonstrate that freshly isolated whole pancreatic islets, selected for size and quality, plated on a plastic surface with fixed laminin-521, non-traumatically adjust their shape to the surface due to their strong affinity to the laminin—the adherent monolayer organoid forms within one week. Islet cell numbers (total and type-specific) were measured by software analysis of the 3D microscopic images. The microscopic 3D imaging and analysis procedures were semi-automated, which made the quantitation of about 200 islets in a 96-well plate assay format feasible.

The monolayer structure of this novel islet culture allows for direct and equal exposure of all the islet cells to experimental drugs/chemicals. Using this novel assay system, we examined the cytotoxic effect of STZ treatment of isolated islets *in vitro*. We showed a dose-dependent cytotoxic effect, especially on β-cells, thus causing an unhealthy shift in α-to-β cell ratio. However, the endocrine power of β-cells did not change by STZ treatment.

### Contribution to 3R strategies

Diabetes is a systemic metabolic disorder; therefore, as a preclinical study, diabetic animals are considered ideal models for diabetes research. However, animal studies need many animals to be sacrificed, are time-consuming and costly, and often specific techniques are required to achieve reliable data. Therefore, for screening new drugs, it will be helpful to establish reliable *in vitro* methods before *in vivo* studies with animal models.

In this study, we showed an islet *in vitro* culture system, a reasonable alternative to diabetic animal research. Below, we will discuss the pros and cons of this method concerning the 3R objectives: (I) reducing the number of animals required for an experimental study, (II) reducing animal suffering (refinement), and (III), if possible, replacing animals with non-animal experimental biologically relevant models.

In our method, 2–3 mice would be enough to get a sufficient number of small islets to make an assay-like experiment, evaluating the dose-dependent effect of an experimental chemical on islet cell populations and function (**Reduction**). In this report, about 200 islets are needed for one set of experiments with five different experimental conditions, and additional staining controls.

In our experimental setup, a small number of healthy mice are euthanized without exposure to stressful or painful procedures. All experimental interventions (STZ intoxication, inflicting islet cell death and metabolic system damage, exposure to low and high glucose levels, insulin-containing sample collection) are performed *in vitro* only. We thereby avoid animal suffering caused by intoxication, severe diabetes symptoms, blood sampling, fasting, injections, and oral gavage. (**Refinement** and **Replacement**)

In addition, our method specifically requires small islets that often remain unused from islet transplantation experiments. Such small islets are usually discarded due to technical reasons. However, the function of small islets is equal to or better than bigger islets [12]. By collaborating with a laboratory that routinely performs islet transplantation experiments, one can utilize the small islet material that is a byproduct of the transplantation experiments for preclinical-oriented drug evaluation. In such a case, there is no need to euthanize any extra mice for the *in vitro* experiment.

### The biological relevance of the monolayer whole adherent islet model

This monolayer adherent whole islet model also possesses good biological relevance to the *in vivo* function, such as representing essential niche features of endocrine islet cells [19,37]. The relevance of functional assays is proven by the ability to secrete insulin in a glucose-dependent manner (in GSIS assay). The “endocrine power” and “endocrine sensitivity” of α-, β-, and δ- islet cells in different experimental conditions can be calculated in this model. The cell populations of insulin, glucagon, or somatostatin-releasing cells could be analyzed by immunofluorescent staining of cells, and each hormone production could be measured by ELISA. Based on these data, the “endocrine power”: the amount of insulin released by β-cell per hour was also calculated from the experiments (Fig 7).

The endocrine cells, such as α-, β-, δ-, stay in close contact with each other in the pancreatic islets, which allows them to fine-tune the metabolic regulation *in vivo* [10,11]. The monolayer adherent whole islet model changes the original 3D architecture, adjusts through adhesive forces, and thus shifts to a flatter shape. Some principal features of the blood capillaries and islet innervation network, responsible for *in vivo* communication with other organ systems, are lost. However, the monolayer allows endocrine cells direct access to oxygen, glucose, and other important molecules while keeping some of the original cell-cell contacts and re-establishing the essential supportive interface with the natural extracellular matrix (laminin-521). Though not identical to the *in vivo* situation, our *in vitro* monolayer adherent whole islet culture system represents many essential features.

Functional quantification using whole islets could be difficult due to the uncertain number of β-cells inside the islets and the different distribution speeds of fluid inside the islets [38]. Our monolayer whole islet model has also been proven to secrete insulin in a glucose-dependent manner in GSIS assay. Moreover, in the current study, the insulin secretion by one β-cell in response to high glucose was calculated as 6–7 pg/β-cell/hour, which is substantially higher compared to freshly isolated primary β-cell *in vitro* culture reported average β-cell productivity of about 0.5 pg/β-cell/30 minutes (which can be roughly approximated to about 1 pg/β-cell/hour) [39]. This observation corresponds well with previous findings by Callewaert et.al. [14], which showed that whole islet dissociation into cell suspension results in about 8 times reduction of “endocrine power”. They concluded that “the amount of β-cells needed for normalization of glycemia was more than eightfold higher when using dispersed cell aggregates versus unmanipulated islets”.

Taken together, though the original islet 3D architecture is rearranged through non-traumatic reformation into a monolayer in our flat islet model, many major features of the endocrine islet cell niches are maintained, i.e., endocrine cell-cell junctions, contact with essential extracellular matrix and excellent access to oxygen, glucose, nutrients in culture medium.

### Technical aspects of the proposed model

We introduce a format for quantitative analysis of about 200 islets that is user-friendly, flexible, semi-automated, and affordable. Freshly isolated whole spherical islets are an excellent research model, but are difficult to translate into a user-friendly quantitative assay format. It is a common problem that isolated spherical whole islets easily detach from the culture plate surface and may get lost in washing steps, causing issues for experiment interpretation. Whole monolayer adherent islets adhere to laminin-521-coated surfaces very strongly. This connection is not compromised even during repeated rounds of liquid handling, such as in GSIS or STZ treatment. Importantly, STZ treatment does not compromise the islet-to-surface connection in the surviving islet cells. Monolayer adherent whole islets, unlike spherical isolated whole islets, are very easy to work with in culture and immunohistochemistry. These adherent islets stay firmly attached to the plastic surface and do not fall off during treatment, which makes it easy to perform experiments correctly and achieve reliable results. Though the method requires accuracy and attention to detail, every procedure stage is simple and does not require refined technical skills or years of special training.

As mentioned in “Materials and Methods,” the method requires massive microscopic imaging and image analysis. However, all the tedious procedures are delegated to automated equipment and software. One of the most time-consuming stages – quantitative analysis of multiple islet 3D images – could be performed at a distance, accessing a core facility’s powerful servers.

This *in vitro* method can be supplementary to a large project, e.g., evaluation of a novel drug treatment before the preclinical stage, dissecting the direct effect of drugs on each endocrine cell type, which could be difficult to reveal using an *in vivo* approach. Our method works as a quantitative semi-automated 96-well plate assay, allowing the extraction of quantitative data regarding cell numbers, total and type-specific, “endocrine sensitivity,” and “endocrine power”.

We have demonstrated how quantification and statistical analysis are possible for a modest setup with five different experimental conditions (including an untreated control). Through titration, the STZ concentration sufficient to induce diabetes-like phenotype *in vitro* was estimated. In our study, 0.6 mM STZ damaged pancreatic islets, and the effect was not reversible afdter one week in a toxin-free culture. It is possible that larger experiments would allow for the capture and evaluation of more subtle effects of lower STZ concentrations.

The dose-dependent diabetogenic effect of STZ has been examined in rodent models [29–31]. The concentration achieved from the *in vitro* data cannot be directly translated to the *in vivo* case because total body mass and metabolic activity affect drug efficacy. However, our model can demonstrate the direct effect of drugs on the endocrine cell level and could reveal mechanisms not easily seen *in vivo*.

### Future perspectives

For drug development, the investigation of optimal drug concentration is necessary. *In vitro* assays allow for an investigation of high-dose toxicity without the need for live animal experiments. We used STZ, which has a small window between effective and toxic doses. For other similar drugs, it would be recommended to find *in vitro* alternatives to animal experimental methods.

Awareness of animal welfare is not the same worldwide, and legislation in many countries allows for large experiments involving unnecessary animal suffering without proper scientific and ethical justification. For the sake of animal well-being, it is therefore important to develop 3R experimental methods that are attractive to researchers and would be chosen voluntarily over animal research. It is often costly to develop a reliable *in vitro* method instead of conventional protocols involving animals; however, once the methods are established, *in vitro* experiments are often more easily reproducible and cost-saving.

Our methods offer an experimental approach that is user-friendly and affordable, and enables us to conduct quality diabetes research. In our study, we captured high-quality islet 3D images and used specific software to analyze the data in collaboration with the local core facility, which owns the equipment for 3D confocal microscopy and provides the service of quantitative analysis of 3D images in batch mode. Such an approach allows us to use high-quality imaging services for a very modest fee, and one is not limited to local-only services. However, it is technically possible to quantify the islet cell numbers by ordinary 2D fluorescent microscopy available in any immunohistochemistry facility (e.g., in hospital laboratories). Since the whole islet culture is a monolayer, the evaluation can be done using a similar method to the one used for the analysis of immunostained tissue slices.

The monolayer adherent whole islet approach is not limited to the *in-vitro*-STZ-induced diabetes research model. The same set of experimental procedures can be used to research many other questions regarding islet biology, such as investigation of regeneration of α-, β-, δ-, PP- or ε- islet cells, drug toxicity/efficacy studies, search for sophisticated combinations of drugs for possible synergistic effect, and many other questions. We noted that the islet monolayer could be cultured for over two months without losing insulin release capacity under glucose stimulation, and cell function could probably be followed for longer than the period demonstrated here. However, careful verification is needed before planning such experiments. Our approach enables easy measurement in a plate-screening setup of simple parameters such as islet cell number (total, α-, β-) and insulin release sensitivity to glucose, as well as essential parameters that are often missed by research, such as α-to-β cell ratio and β-cell “endocrine power”.

## Conclusions

1. In conclusion, we propose a monolayer adherent whole islet *in vitro* culture system. The method is based on the entire islet non-traumatic reformation *in vitro* due to its strong affiliation to its natural ligand, laminin-521. The islets remain whole, and the cells and cell-cell junctions are not disrupted.
2. The monolayer allows direct access of experimental molecules in the culture medium to all the islet cells.
3. Using this model, the dose-dependent effect of STZ on pancreatic islet cells was examined to demonstrate how this model can be used for drug toxicity monitoring and quantifying cell function.
4. The proposed experimental approach is 3R-friendly, versatile, adaptive, user-friendly, semi-automated, and affordable for small research groups with limited funding.

## Supporting information

S1 Fig Microscopical analysis of isolated/cultured pancreatic islet cells. **(A) Heterogeneity of pancreatic islet cells.** The whole isolated mouse islet *in vitro* culture was cultured on the uncoated plastic surface and was non-attached. Phase contrast microscopy, magnification 20x. **(B) Central necrosis during culture.** The whole isolated mouse islet *in vitro* culture was cultured on a laminin-521-coated plastic surface for about 2 weeks. Phase contrast microscopy, magnification 20x. Laminin-521, a natural ligand for islet cells, allows the anchoring of whole islets to the surface firmly and thus promotes whole islet attachment, spreading, and flattening. Though the islet is attached and flattened, signs of central necrosis are visible in the central part (dark area), while excellent viability and spreading are seen on the islet borders. **(C) Overgrown fibroblastic-like cells.** The whole isolated mouse islet *in vitro* culture was contaminated with non-islet cells and cultured on a laminin-521 coated plastic surface for about 2 weeks. Immunofluorescent staining: blue: DAPI (all cell nuclei), green nuclei: EdU ClickIt EdU Alexa Fluor-488 imaging kit, Invitrogen, #C10337 (marker of proliferation; due to overexposure may look white-green, red: CellMask™ Deep Red Plasma membrane stain, Invitrogen, #C10046 (indicates all cells, even very flat and spread fibroblast-like cells). The image contains a compilation of 3x3 adjacent fields, captured by spinning disc confocal microscopy, magnification 20x. **(D) Flat but Multiple layers of pancreatic islet cells.** The whole isolated mouse islet *in vitro* culture was cultured on a laminin-521-coated plastic surface for about 2 weeks. Phase contrast microscopy, magnification 20x. Bulging is visible in the central part of a flat whole islet spread on laminin-521-coated plastic. Due to the bulging, access of experimental molecules from the culture medium to the central cells in multilayered islets is restricted.
(TIF)

S1 File Insulin concentrations in glucose-stimulated insulin release test, and Imaris calculated islet cell numbers; data for Figs 4, 6, and 7. (XLSX)
